# Ecosystem functions in degraded riparian forests of southeastern Kenya

**DOI:** 10.1002/ece3.8011

**Published:** 2021-08-25

**Authors:** Jan Christian Habel, Werner Ulrich

**Affiliations:** ^1^ Evolutionary Zoology Department of Biosciences University of Salzburg Salzburg Austria; ^2^ Department of Ecology and Biogeography Nicolaus Copernicus University Toruń Toruń Poland

**Keywords:** arthropods, drone, habitat destruction, invasive plant species, land‐use, *Lantana camara*, pollination, predation, seed dispersal

## Abstract

Species community structures shape ecosystem functions, which are mostly stronger pronounced in intact than in degraded environments. Riparian forests in semiarid Africa provide important habitats for endangered plant and animal species and provide various ecosystem functions, that is, services to people settling along these streams. Most of these riparian forests are severely disturbed by human activities and dominated by invasive exotic plant species in the meanwhile. Thus, ecosystem functions are negatively influenced. While most studies have analyzed a specific metric to measure the degree of ecosystem function, little is known about how strongly different ecosystem functions respond to anthropogenic disturbances in parallel. In this study, we analyzed a set of four proxies of ecosystem functions, ground‐dwelling arthropod abundances, pollination, seed dispersal, and predation, along a highly disturbed riparian forest in southeastern Kenya. We assessed the land cover and land use manually and with an Unmanned Aerial Vehicle. Our data show that ecosystem functions respond differently to vegetation cover, human disturbances, and the availability of the invasive exotic shrub *Lantana camara*. The occurrence of representatives from the groups Saltatoria and Formicidae profits from heterogeneous habitat structures and natural riparian forest, while representatives of the Araneae profit from high proportion of agricultural fields. In general, predation is higher in mixed land use and natural riparian forest, while pollination and seed dispersal showed no significant trend in regard on land coverage. Along with this, predation also increased with rising proportion of natural riparian forest, while the proportion of agricultural land negatively affects predation, but in parallel showed a slightly significant positive trend with seed dispersal. Human disturbances and the occurrence of the invasive exotic *L*. *camara* shrub did not significantly affect our metrics of ecosystem functioning, except of the negative impact of human disturbances on pollinators. In conclusion, our results underpin that ecosystem functions respond highly variable and individually to environmental changes.

## Graphical abstract

Ecosystem functions are crucial for human well‐being. We analyzed four proxies to evaluate ecosystem functions in a degraded riparian forest in southeastern Kenya. Our data indicate that intense land use and the invasion of exotic plants negatively impact some of the ecosystem functions assessed.

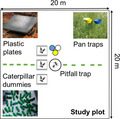

## 1 INTRODUCTION

The transformation of natural and near‐natural habitats into anthropogenic, that is, intensively used landscapes, such as settlements, agricultural fields, pastures, and plantations, has been ranked as the top driver causing global biodiversity loss (Maxwell et al., 2016; Sala et al., 2000). In addition, the devastation and degradation of habitats influence biodiversity loss significantly due to biotic homogenization (Olden et al., 2004). Studies have shown that ecosystem functions occur at a significantly higher rate in heterogeneous and intact landscapes, while functions occur at a significantly reduced rate in homogeneous and/or degraded habitats (Winqvist et al., 2011).

Ecosystem functions provided by nature are manifold and integrate abiotic (e.g., water, soil, air) and biotic (e.g., pollination, predation) factors, and take place at the global, regional, and local scale (Hooper et al., 2005). Previous studies showed that human activities disturbing ecosystems directly modify abiotic and biotic interactions, and subsequently species community structures and ecosystem functions (Felipe‐Lucia et al., 2020). For example, extensive deforestation might significantly change climatic conditions at the regional scale (Lawrence & Vandecar, 2015) and thus erode this ecosystem function. Pollination activity is strongly reduced in areas with intense agricultural activities (Tscharntke et al., 2005). Furthermore, habitats dominated by one invasive exotic plant species frequently provide less ecosystem functions when compared with still intact and diverse environments (Baude et al., 2019; Linders et al., 2019).

Human well‐being directly relies on various ecosystem functions, that is, services (Daily, 1997). These services can be grouped into provisioning services (goods produced or provided by ecosystems), regulating services (benefits from regulation of ecosystem processes), supporting services (factors necessary for producing ecosystem services), and cultural services (nonmaterial benefits from ecosystems; Millennium Ecosystem Assessment, 2005). Economies, such as the agricultural sector, strongly rely on ecosystem services, such as plant pollination by insects (Klein et al., 2007) and predation (i.e., pest control) across agricultural fields (Tschumi et al., 2018). Intact ecosystems with high levels of ecosystem functions may significantly increase the yields of food crops and thus support food security and positively influence human well‐being (Power, 2010).

The majority of people living in sub‐Saharan Africa conduct and strongly rely on subsistence agriculture. Thus, intact ecosystems and landscapes with respective provisioning services form the basic prerequisite of human well‐being in the rural areas of sub‐Saharan Africa (Cardinale et al., 2012; Hooper et al., 2005). However, a major part of the landscapes in sub‐Saharan Africa suffers under in‐appropriate land use, weak or lacking land management, and extreme demographic pressure with subsequent increasing agricultural intensity to feed the growing human population (Habel et al., 2015). As a consequence, soil fertility is decreasing, ground water levels lowering, and ecosystem integrity and biodiversity affected negatively across major parts of East Africa (Rukundo et al., 2018).

A very important habitat and settlement area are the gallery forests along streams in the semiarid regions of East Africa. These gallery forests provide valuable habitats for numerous endangered animal and plant species. At the same time, these riparian strips are also coveted settlement areas for people who benefit from the various ecosystem services provided by the rivers and the surrounding forests. This creates a conflict between conservation and the (over)use of these resources. In consequence, today, most gallery forests are highly disturbed or have been destroyed completely. This also applies to the Nzeeu River, a small stream located in southeastern Kenya. This area suffers under extreme demographic pressure and high poverty rates, deforestation, and subsequent devastation of the ecosystems by invasive exotic shrub species *Lantana camara* (Habel et al., 2018). To study the impact of land use and land devastation on ecosystem functions (i.e., services), we measured a set of various ecosystem functions for study plots established in a degraded riparian forest ecosystem along Nzeeu River. In total, we assessed four proxies of ecosystem functions, which are all crucial to people and food production, namely, aboveground secondary productivity (arthropod abundance), pollination, predation, and seed dispersal. For each study plot, we also measured land and plant cover, particularly the presence of the exotic invasive *Lantana camara* shrub species, and the degree of human disturbance. Based on these data, we will answer the following research questions:

1. How do human induced changes in land use affect taxonomic diversity and ecosystem functioning?
2. To what degree do invasive species alter these functions?
3. Do these changes vary with the degree of disturbance?

## 2 METHODS

### 2.1 Study area

The Nzeeu River with adjoining dryland savannahs and agricultural fields is located south of Kitui city in southeastern Kenya (Appendix S1). This river with remnants of riparian forest provides important ecosystem services to local people settling along this stream, such as water/groundwater for water irrigation, timber for house construction, and wood as an energy source for cooking (Habel et al., 2018; Teucher et al., 2015). A major proportion of the original and diverse riparian forest has been cleared and converted into agricultural land (mainly fields of maize and sorghum). In the wake of these habitat transformation and disturbance, the invasive exotic shrub *Lantana camara* invaded extensively across East Africa (Njoroge & Bennun, 2000) as well as along Nzeeu River (Habel et al., 2018; Schmitt et al., 2019; Teucher et al., 2015). This exotic shrub is known to be highly expansive (Prasad, 2012) and has expanded since the colonial era, when this plant species was introduced in many African countries for fodder, energy, and ornamental purposes (Day et al., 2003; Kannan et al., 2013; Urban et al., 2011). In the meanwhile, this species has become a pest, particularly in already disturbed habitats (Duggin & Gentle, 1998; Foxcroft et al., 2010; Vardien et al., 2012).

### 2.2 Rapid ecosystem function assessment (REFA)

We set 90 study plots (squares of 20 × 20 m each) with 45 plots at each side along Nzeeu River. Each of these study plots was located at least 100 m distant from the river to avoid potential overflooding, and at least 100 m distant between each other to minimize potential effects from autocorrelation. We used a standardized, low‐tech, and easily repeatable technique to measure four proxies of ecosystem functions (see Meyer et al., 2015): aboveground secondary productivity (arthropod abundance), pollination (of field crops), predation (of pests), and seed dispersal. Data collection was performed after the rainy season in March (14–31) in the year 2016.

**FIGURE 1:**
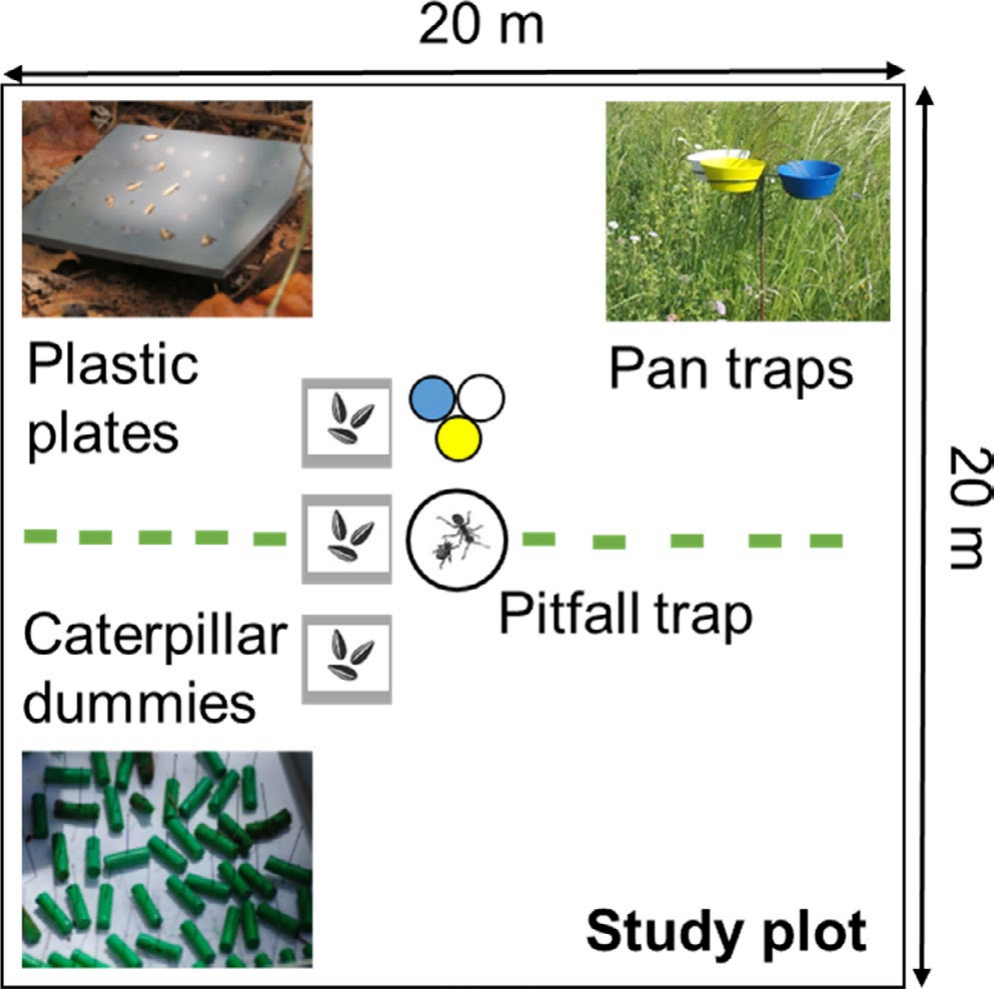
Study plot set perpendicular to Nzeeu River. In the center, there is the pitfall trap and the three pan traps (blue, yellow, white). The three seed plates are 3 m apart from each other, and 10 artificial caterpillar dummies were placed on the floor at least 1 m away from one another

The amount of arthropods represents the level of aboveground secondary productivity (Ebeling et al., 2018). Thus, arthropod biomass significantly influences the stability and functioning of ecosystems. We conducted standardized suction sampling of invertebrates (see Southwood & Henderson, 2009). To capture arthropods we set one pitfall trap (plastic cups with 7 cm opening and 15 cm height, filled with diluted dishwasher) in each study plot for six days (see Figure 1). To avoid zero inflation, for the present study we used only those taxa found in more than half of the 90 plots and being represented by more than 100 individuals: Araneae (69 plots; 412 individuals), Coleoptera (46; 213), Heteroptera (55; 453), Formicidae (Hymenoptera) (80; 21,890), and Saltatoria (Orthoptera) (58; 312). These different taxonomic levels were chosen specifically to accomplish the >100 individual criterion. These groups represent different ecological and foraging habits. For example, representatives of the group Araneae and Formicidae live predominantly predatorily and frequently predate other insects, representatives of the group Coleoptera and Heteroptera live partly herbivorously in vegetation structures but also predatorily in open habitats, and representatives of the group Saltatoria live mainly herbivorously. Therefore, different land cover and potential human disturbance could have very different effects on these groups with different behaviors. We dried the material in heat chambers for 10 days at 40°C. We then determined the dry weight. All raw data from these assessments are compiled in Appendix S2.

We measured pollination based on the total number of insects caught with yellow, white, and blue pan traps. Several studies have shown that these colors exhibit the highest sampling efficiencies across a wide array of different taxa of flying insects (Campbell & Hanula, 2007; Nuttman et al., 2011; Westphal et al., 2008; Wilson et al., 2008). Pan traps were filled with dish washer dilution and placed at a height of 1 m. Three traps were positioned in the center of each study plot (see Figure 1). Traps were activated from 7 a.m. until 5 p.m. per day, and over a period of 6 days. We subsequently dried the material collected in a heat chamber for 10 days at 40°C and weighted the material after drying.

Seed dispersal by animals is of high relevance in many ecosystems, as animals that move seeds from source plants are driving plant gene flow and population dynamics in habitats, as well as vegetation recovery in degraded landscapes (Kremen et al., 2007). We conducted seed dispersal, that is, seed removal experiments by using three gray 10 × 10 cm^2^ plastic plates on which we placed 25 sunflower seeds on each plate. All 25 seeds were cut into half to avoid potential germination of the seeds (Vander Wall et al., 2005). The three plates were placed parallel to the river, with three meter spacing from each other (see Figure 1). We counted the number of seeds remaining on each plate after 60 min. This experiment was conducted from 7 a.m. to 5 p.m., and over a period of 6 days.

Natural pest control may significantly increase agricultural yields. We measured the level of pest control by measuring predation rates. For this, we counted attacks on artificial caterpillars made out of green plasticine (Koh & Menge, 2006; Loiselle & Farji‐Brener, 2002; Ruiz‐Guerra et al., 2012). This method allows to differentiate among predator groups (in our case, we differentiated among insects, rodents, birds, and snails) by respective bite marks on the green plasticine (Howe et al., 2009). We used ten 2‐cm‐long caterpillar dummies for each study plot. Dummies were set on the ground to the left and the right of each pitfall trap (see Figure 1). We exposed these dummies for 24 hr to attract diurnal as well as nocturnal predators. Subsequently, we assessed all bite marks and calculated the proportion of dummies with at least one bite mark. As one predator may cause several bite marks, we did not consider bite frequency per dummy. Vanished dummies were classified as predated (without any further information on the group of predator; Meyer et al., 2017).

### 2.3 Environmental parameters

For each 20 × 20 m study plot, we estimated the land cover (in percentage) by considering the following categories: grass, herbs, native shrubs, invasive shrubs (*L*. *camara*), trees, bare soil, and agricultural land. In addition, we assessed the degree of direct and punctual human disturbance considering timber extraction, signs of fire, and grazing. We divided the degree of disturbance into three categories: no human disturbance, medium human disturbance, and high human disturbance. We considered the following parameters: timber extraction, signs of fire, and grazing. Estimates on land cover and human disturbances were assessed for all plots by the same person and were collected when rapid ecosystem function assessment was performed.

In addition, we collected land cover data using an unmanned aerial vehicle UAV (DJI Phantom 2 drone) equipped with an orthogonal attached RGB digital camera GoPro HERO 4 Black (GoPro, Inc., San Mateo) mounted on a Zenmuse H3‐3D gimbal (details on the procedure of data collection are provided in Habel et al., 2018). The attached digital camera was configured using medium resolution settings of seven megapixels and focal length of 21.9 mm equivalent, resulting in picture dimensions of 2,250–3,000 px, with aspect ratio of 3–4 to reduce fish‐eye distortion. The aerial photographs were subsequently assembled with the AgiSoft Photoscan Professional software (Agisoft, 2016) using medium‐quality dense cloud processing and mesh construction settings. Based on sufficient ground control points which were taken in the field, processed imagery was exported as orthomosaic into geotif raster files with geometric accuracy below 1.97 m (1.00 m in longitudinal error, 1.38 m latitudinal error, and 0.99 m altitudinal error). The tiled orthophotos were subsequently mosaicked using gdal‐function merge in QGIS (GDAL 2015) and prepared for further analysis.

A land cover map raster file was created using Image Pyramids with the software QGIS Development Team (2016). We set 40 m buffers around each of the 90 study plots. The following land cover categories were digitized as polygons: trees, shrubs, open agricultural land, and riverbed. Roads and paths were digitized as lines. Proportion of land cover types (identical with the ones above) were calculated by intersecting the 40 m buffers around each study plot with digitized land cover vector data. The proportional area of each land‐use category was calculated as the area of a certain land‐use category within the intersect layer divided by the total area covered by the 40 m buffer.

### 2.4 Statistics

For the landscape analyses, we used the coverage of *L. camara*, the proportion of agricultural fields, and the proportions of land cover of herbs, crops, trees, shrubs, bare soil, and agricultural land. These variables were only moderately correlated (Appendix S2). The dominant eigenvector of the dissimilarity matrix (Gower dissimilarities) of the land cover proportions served as an estimate of the variability of plant cover among the study plots.

We used fixed effects generalized linear modeling to link pollination, seed dispersal, and predation as response variables (separate models for each variable) to the degree of human disturbance and the proportion of *L*. *camara* coverage (metric variables) and to land cover types (fixed effect). As the predictors include zero counts, we used a Poisson error structure and an identical link function. Goodness of fit was based on Wald statistics. The study plots were spatially nonindependent. To avoid biases in the estimation of parametric significances, we used eigenvector mapping and calculated the dominant eigenvector of the geographical Euclidean distance matrix (PCA1) that covers the spatial distribution of plots. We added PCA1 as an additional covariate to the linear models.

## 3 RESULTS

**FIGURE 2:**
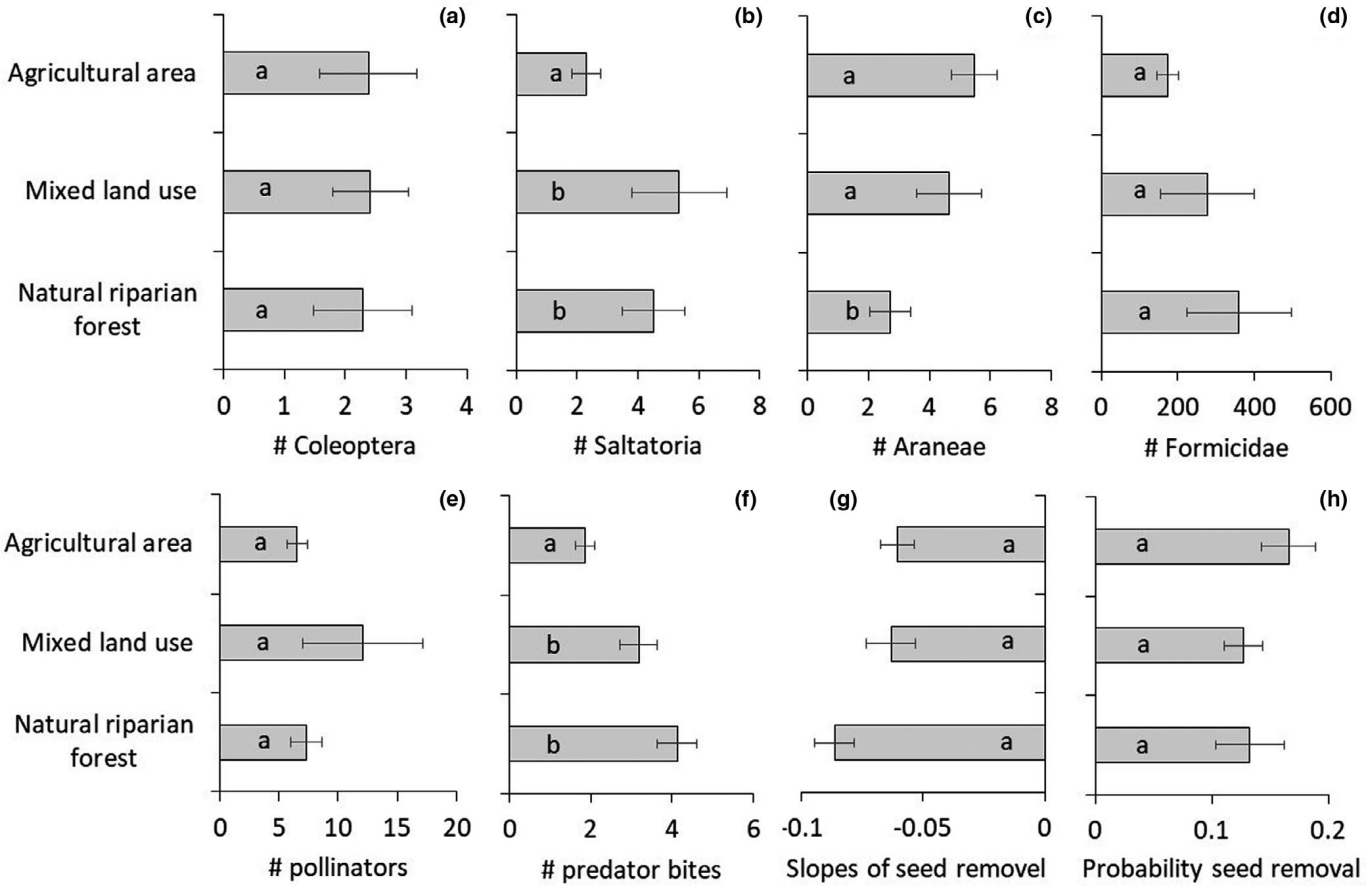
Mean numbers (#: numbers of) of important arthropod taxa collected in pitfall traps (a–d), of pollinators in pan traps (e), of predation (numbers of bites, f), and of average slopes (g) and probabilities of seed removal (h) in the three different land use types. Farmland *N* = 49, natural vegetation *N* = 24, mixed land use *N* = 17. Error bars denote one parametric standard error. Bars not significantly different at *p* < .05 are marked with identical letters

We found taxon‐specific response of arthropods to differences in land cover, human disturbance, and the occurrence of the invasive shrub species *L. camara* (Table 1). High proportions of farmlands were negatively associated with the abundance of Saltatoria (Figure 2b) and ants (Figure 2d), but positively associated with the abundance of spiders (Figure 2c). These contrasting relationships demonstrate that total plant cover had only marginal influence on total arthropod abundance (Table 1).

**TABLE 1:** Generalized linear modeling (*N* = 90) using land use (categorical), plant cover, human impact, *Lantana* occurrence, sky cloud cover, and the dominant eigenvector (PCA1) of the geographical Euclidean distance matrix as predictors for the abundance of important arthropod taxa

Variable	*df*	Coleoptera		Hemiptera		Saltatoria		Formicidae		Araneae	
		Wald value	Parameter	Wald value	Parameter	Wald value	Parameter	Wald value	Parameter	Wald value	Parameter
Land use	2	2.08		3.02		6.45*		7.02*		1.16	
Plant cover	1	0.10	0.01	1.19	−0.05	1.28	−0.03	0.31	−0.94	3.61^+^	−0.06
Human disturbance	1	7.37**	0.03	4.35*	0.12	<0.01	<0.01	2.71^+^	3.53	7.87**	0.10
*Lantana* occurrence	1	1.33	−0.02	1.37	−0.06	19.82***	−0.09	3.88*	−3.68	1.13	−0.04
Cloud cover	1	0.07	−0.01	0.46	0.04	0.85	−0.03	0.38	1.50	0.55	−0.03
PCA1	1	19.28***	10.27	0.13	4.28^+^	0.56	2.89	1.30	349.9	1.32	6.21

Note

Parametric significances ^+^ *p* < .10, *p < .05, **p < .01, ***p < .001.

After correcting for covariates, the degree of human disturbance positively influenced the abundance of Coleoptera, Hemiptera, Formicidae, and Araneae (Table 1). Total arthropod and pollinator abundance showed no significant differences among habitats with different degrees of natural vegetation (Figure 2a,b). In turn, the occurrence of *L*. *camara* showed a significantly correlated with arthropod abundance (Table 1).

**FIGURE 3:**
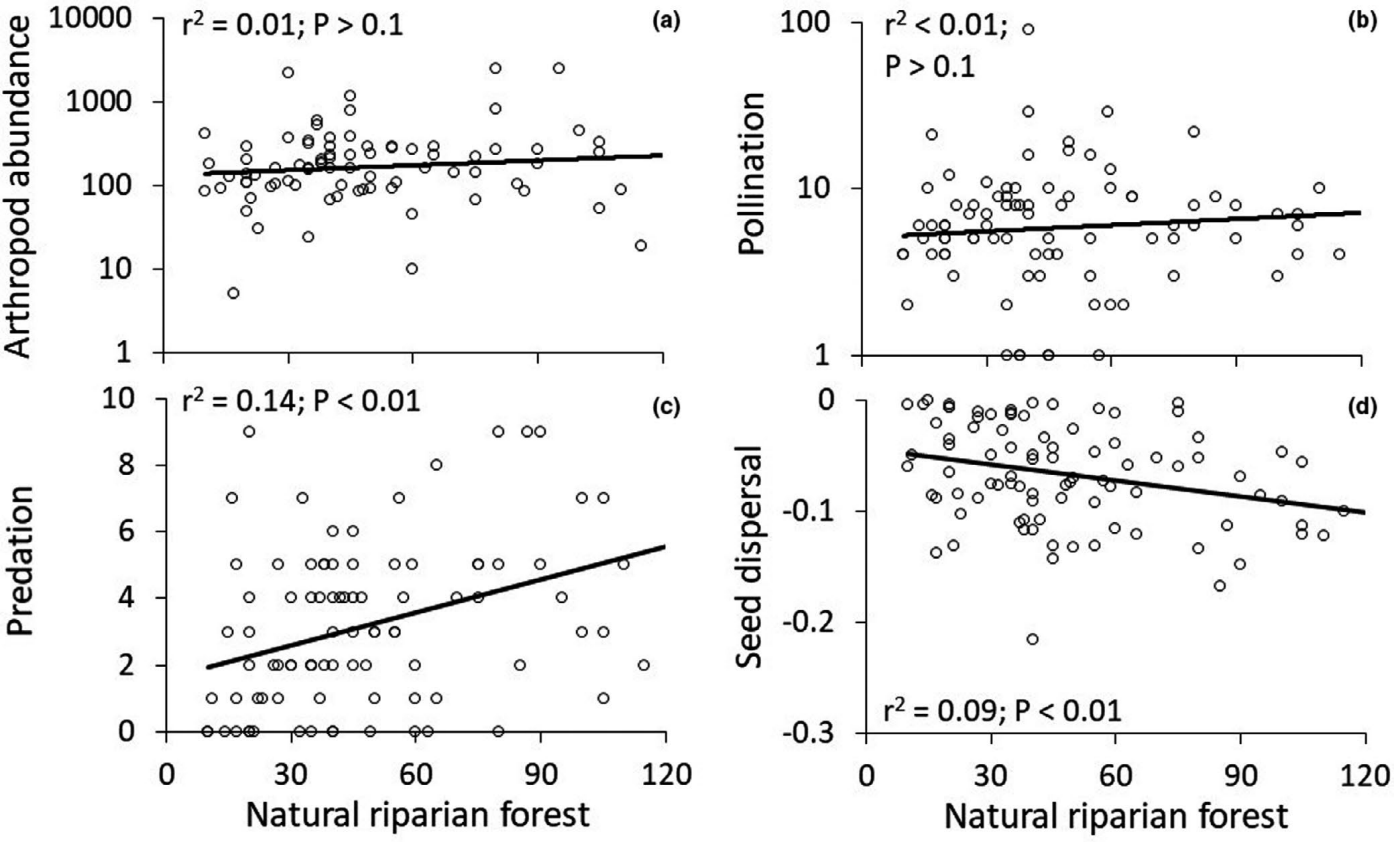
Dependency of total arthropod abundance (a), pollinator abundance (b), predator pressure (c), and the strength of seed dispersal (d) on the proportional of native plants in 90 sample plots. Parametric significances and *r* ^2^ values refer to ordinary linear regressions

**FIGURE 4:**
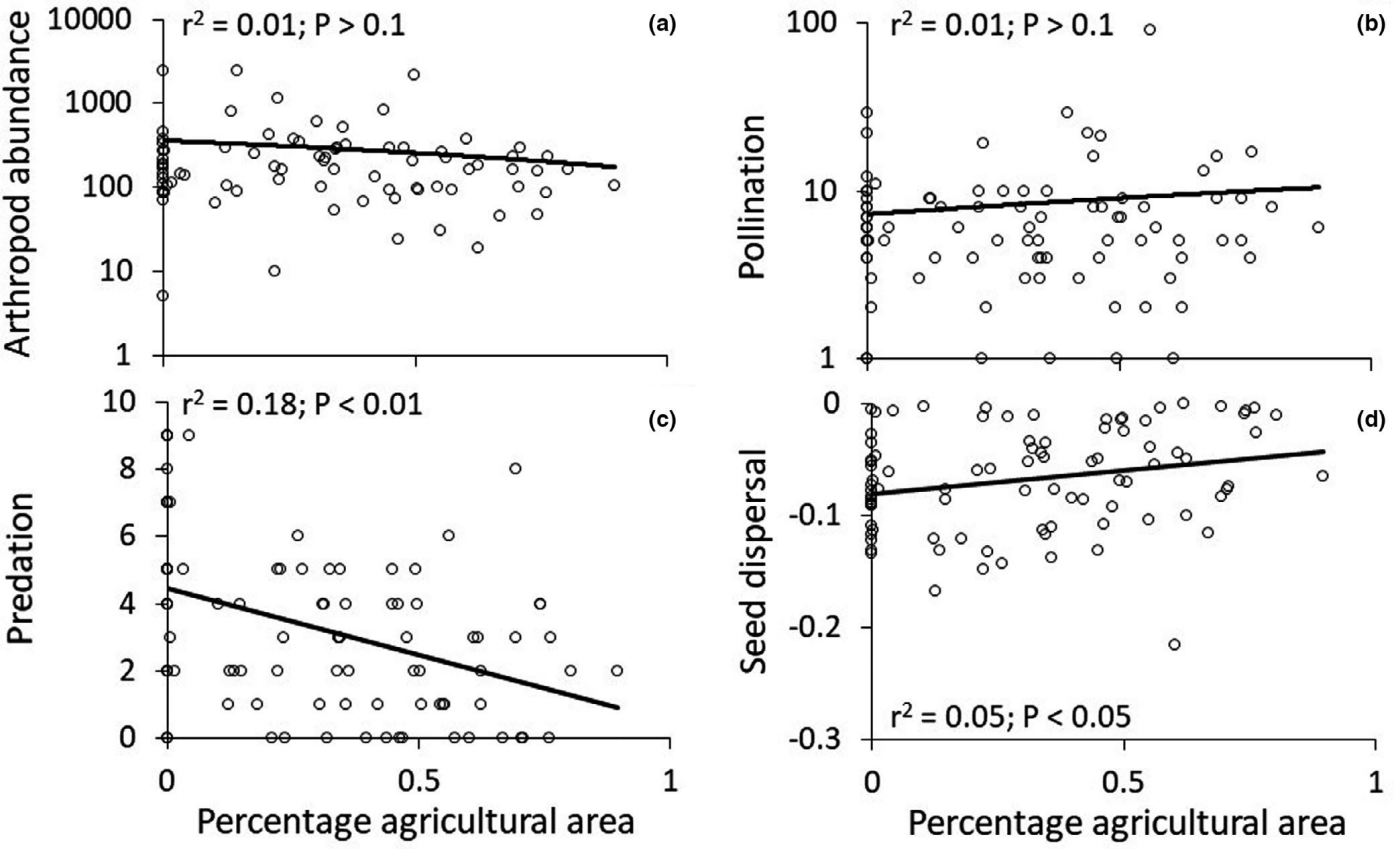
Dependency of total arthropod abundance (a), pollination (b), predation (c), and seed dispersal (d) on the percentage of open agricultural land in the buffer zone. Parametric significances and *r* ^2^ values refer to ordinary linear regressions

Land cover significantly influenced pollinator abundance and pollinator weight and predation pressure (Table 2). Predation pressure (almost exclusively performed by insects) was lower at sites with a high proportion of agricultural land (Figure 3f) and increased with the proportion of natural riparian forest (Figure 3c). Seed removal increased with the proportion of natural riparian forest (Figures 3g and 4d). Human disturbance and the occurrence of the invasive exotic *L*. *camara* shrub significantly affected most of our metrics of ecosystem functions (Table 2).

**TABLE 2:** Generalized linear modeling (*N* = 90) using land use (categorical), plant cover, human impact, *Lantana* occurrence, sky cloud cover, and the dominant eigenvector (PCA1) of the geographical Euclidean distance matrix as predictors for important ecosystem functions

Variable	*df*	Pollinator abundance		Pollinator weight		Seed dispersal		Insect predation pressure		Vertebrate predation pressure	
		Wald value	Parameter	Wald value	Parameter	Wald value	Parameter	Wald value	Parameter	Wald value	Parameter
Land use	2	4.06^+^		7.95*		0.03		5.02^+^		7.48*	
Plant cover	1	0.03	0.01	1.21	0.10	0.22	<0.01	0.02	<0.01	0.51	0.01
Human disturbance	1	0.27	−0.03	5.95*	−0.34	0.03	<0.01	1.07	−0.01	2.28	−0.02
*Lantana* occurrence	1	2.86^+^	0.09	2.25	−0.17	0.04	<0.01	0.13	<0.01	0.08	0.00
Cloud cover	1	0.21	0.03	1.73	−0.21	0.06	<0.01	5.86*	0.03	1.07	0.02
PCA1	1	2.65^+^	13.19	5.01*	44.95	0.01	−0.02	2.67^+^	2.76	0.42	1.24

Note

Parametric significances ^+^ *p* < .10, *p < .05.

When taking the larger 40 m radius buffer zone into consideration, we found a significant positive influence of open agricultural land on the abundance of Coleoptera, Hemiptera, and Araneae, while Saltatoria were negatively affected (Table 3). However, total insect abundance did not significantly correlate with the percentage of open agricultural land (Figure 4a). Except for the positive influence of tree coverage on spider abundance, shrubs were not significantly related to insect abundance (Table 3). We also found a positive effect of farmlands and negative influences of the occurrence of trees on pollinator abundance (Table 4, Figure 4b). Total pollinator weights were negatively correlated with tree and shrub coverage within the 40 m radius buffer zone, while open agricultural land did not significantly influence the total weight of biomass from flying pollinators (Table 4). Insect predation pressure decreased with increasing proportions of agricultural land and with increasing shrub coverage (Table 4, Figure 4c). In turn, buffer zone habitat types did not significantly influence seed removal (Table 4, Figure 4d).

**TABLE 3:** Generalized linear modeling (*N* = 90) using open farmland area, tree cover, shrub cover, and the dominant eigenvector of the geographical Euclidean distance matrix (PCA1) as predictors for the abundance of important arthropod taxa

Variable	*df*	Coleoptera		Hemiptera		Saltatoria		Formicidae		Araneae	
		Wald value	Parameter	Wald value	Parameter	Wald value	Parameter	Wald value	Parameter	Wald value	Parameter
Open farmland	1	13.43***	0.002	6.86**	0.003	5.37*	−0.002	0.07	0.04	41.08***	0.005
Tree area	1	1.42	<0.001	2.06	<0.001	0.03	<0.001	1.34	0.23	19.36***	0.004
Shrub area	1	0.46	<0.001	2.26	<0.001	1.02	<0.001	0.84	0.15	2.77	0.001
PCA1	1	54.99***	10.53	1.23	−2.510	1.39	2.270	1.27	460.10	0.01	−0.17

Note

Parametric significances ^+^ *p* < .10, *p < .05, **p < .01, ***p < .001.

**TABLE 4:** Generalized linear modeling (*N* = 90) using open farmland area, tree cover, shrub cover, and the dominant eigenvector (PCA1) of the geographical Euclidean distance matrix as predictors for important ecosystem functions

Variable	*df*	Pollinator abundance		Pollinator weight		Dispersal		Insect predation pressure		Vertebrate predation pressure	
		Wald value	Parameter	Wald value	Parameter	Wald value	Parameter	Wald value	Parameter	Wald value	Parameter
Open farmland	1	12.58***	0.01	0.34	<0.001	0.23	<0.001	8.92**	−0.01	0.36	<0.001
Tree area	1	4.87*	−0.02	32.44***	−0.01	1.17	<0.001	0.41	<0.001	3.28	<0.001
Shrub area	1	2.88	<0.001	25.14***	−0.01	1.29	<0.001	0.43	<0.001	7.22**	0.01
PCA1	1	33.56***	16.13	6.98**	9.47	39.72***	−0.34	4.73*	3.63	<0.001	−0.14

Note

Parametric significances *p < .05, **p < .01, ***p < .001.

## 4 DISCUSSION

### 4.1 Aboveground secondary productivity

We found that the abundance of the predominately phytophagous Saltatoria and pantophagous Formicidae showed negative associations with open agricultural land, while predatory spiders showed positive associations with agricultural activity. Other studies reported similar contrasting correlations. Lemessa et al. (2015) showed that arthropod diversity differs significantly among gardens in Ethiopia, and conclude that different land‐use types create variations in biodiversity. In our case, microclimatic conditions, ecosystem structures, and resource availability found in natural riparian forest remnants, as well as in exotic *L*. *camara* shrubs, might provide suitable habitats for phyto‐ and pantophages organisms. In contrast, predatory spiders often profit from open heterogeneous agricultural land. This precondition might favor successful predation of other arthropods, such as midges and moths (Grill et al., 2005). However, a more detailed look on the diversity of arthropod species underlines that disturbed ecosystems dominated by some few invasive exotic plant species lead to severe reductions of species richness of herbivorous arthropods (Habel et al., 2018). A reduction of species richness in ecosystems dominated by exotic plant species was already previously reported (Dobhal et al., 2001; Singh et al., 2014). However, its impact on ecosystem functioning, that is, services, is still debated (Devine & Fei, 2011; Pejchar & Mooney, 2009).

We found that human disturbances (such as timber extraction, signs of fire, and grazing) also positively affected some arthropod groups, and some were not positively affected. For example, representatives of Coleoptera, Hemiptera, Formicidae, and Araneae responded positively to human activities (Table 1). Such human disturbances as found across subsistence agricultural fields may even create important habitats for those taxa. The cutting of trees may produce dead wood, and fire and grazing keep open ecosystems that would become overgrown by vegetation succession without such disturbances and produce very important microhabitats for many insects (Schowalter, 2012). In parallel, these disturbances also provide a trade‐off of biodiversity acceleration and the probability of invasion of exotic plant species (see Hobbs & Huenneke, 1992).

### 4.2 Pollination, predation, and seed dispersal

Our study revealed positive relationships between predation and higher proportions of natural riparian forest, but negative between seed dispersal and the proportion of natural riparian forest. Pollination showed no significant trend but provided highest values for plots representing a mix of land use (mosaic of agricultural fields and natural riparian forest). We interpret this finding as an indication that heterogeneous landscapes are functionally superior to homogenized ones. Studies have shown that biotic homogenization has a negative impact on biodiversity and ecosystem functions (Olden et al., 2004). Devastation and biotic homogenization through the expansion of exotic invasive plant species play a central role. However, in order to investigate a concrete effect of biotic homogenization through the spread of *L*. *camara*, it would be necessary to compare an ecosystem that is still natural and one that has been invaded by the alien plant species.

Previous studies have shown a generally positive correlation between the proportion of natural vegetation and ecosystem functions (Campbell & Hanula, 2007; Saunders et al., 2013). Candidates for seed dispersal (mainly conducted by birds) frequently forage through hedges and shrubs, and along trees, and thus directly rely on hedgerows and similar dense vegetation. Therefore, the planting of natural vegetation such as patches of riparian forest can support this ecosystem function. Our data showed that predation was mainly conducted by insects, most probably by representatives of the group Formicidae, the most common representatives of invertebrates in our study area (and the tropics in general). Our findings are in line with other studies showing that natural vegetation support higher predation rates (Meyer et al., 2019). Again, this finding supports the view that heterogeneous natural vegetation is also functionally superior.

Pollination did not show a significant relationship with the amount of natural vegetation, but did show highest abundances for plots with mixed land use (i.e., a combination of agricultural land, gardens, and natural vegetation). Pollinators rely on both natural vegetation (e.g., for larval development) and the availability of nectar sources as important energy source. Previous studies underline that pollinators accumulate in heterogeneous ecosystems, such as gardens and diverse and extensively used agricultural landscapes (Nuttman et al., 2011; Winfree et al., 2007). Thus, in our study, pure natural riparian forest provides similar levels of abundances as pure agricultural land. Pollination, predation, and seed dispersal are important ecosystem services to people, as they support ecosystem stability and may accelerate yields of food crops (Klein et al., 2007; Landis et al., 2000). Thus, patches of natural vegetation, flowering plants, and gardens interspersed in agricultural land may stabilize ecosystems and agricultural systems, accelerate agricultural yields, and subsequently improve human livelihood quality (Habel & Ulrich, 2020; Sutter et al., 2018).

We found that natural vegetation cover positively impacted some of the ecosystem functions measured. Previous studies showed that natural habitats or extensively used ecosystems with flowering plants support both, biodiversity and ecosystem functions, which positively spill over into adjoining agricultural land (Calvet‐Mir et al., 2012; Habel & Ulrich, 2020; Klein et al., 2003, 2007; Ricketts, 2004; Tscharntke et al., 2008) and positively influence human livelihood quality. Thus, conserving the last remnants of natural riparian forest and the planting of indigenous trees and shrubs throughout semiarid agricultural landscapes supports biodiversity and ecosystem functions, that is, services. Our study shows that different arthropod groups and ecosystem functions respond differently to landscape cover and anthropogenic use and disturbance. The ecology and behavioral biology of the respective taxa as well as by which species groups the respective ecosystem function is carried out plays a central role here. We would like to close our contribution by highlighting various caveats of this study, which needs to be considered when interpreting our results.

### 4.3 Caveats of the study

We found no significant effects of landscape configuration on the ecosystem functions studied. This could be related to the small‐scale heterogeneity of the study landscape. In order to detect an effect of landscape on local ecosystem functions, it would be useful to compare two different landscapes, for example, a seminatural landscape and an intensively managed landscape. Furthermore, in this study we did not consider in detail the change in species communities with species‐specific functions. Even if there are differences between species communities depending on disturbance and land use, the functions of different species and species groups could be substituted. Thus, despite potential losses of species richness and shifts in species community composition, we could not detect an effect on ecosystem function (Habel & Ulrich, 2020).

## CONFLICT OF INTEREST

There exist no conflict of interest.

## AUTHOR CONTRIBUTIONS

**Jan Christian Habel:** Conceptualization (equal); Data curation (equal); Methodology (equal); Project administration (equal); Supervision (equal); Validation (equal); Writing‐original draft (equal); Writing‐review & editing (equal). **Werner Ulrich:** Data curation (equal); Formal analysis (equal); Funding acquisition (equal); Methodology (equal); Software (equal); Supervision (equal); Validation (equal); Visualization (equal).

## Supporting information

Appendix S1

Appendix S2

## Data Availability

All raw data of this study will be provided as electronic appendix and thus will be online available to everybody.
