# Supplementary material for: Ecosystem functions in degraded riparian forests of southeastern Kenya
Source: Ecol Evol. 2021 Aug 25;11(18):12665–75. doi: 10.1002/ece3.8011 (PMC8462158; doi:10.1002/ece3.8011)

**Supplementary material appendix S1:**

**Ecosystem functions in degraded riparian forests of south-eastern Kenya**

Jan Christian Habel^1^ & Werner Ulrich^2^

^1^Evolutionary Zoology, Department of Biosciences, University of Salzburg, A-5020 Salzburg, Austria

^2^Department of Ecology and Biogeography, Nicolaus Copernicus University Toruń, 87-100 Toruń, Poland

Study area (star in smaller maps) and an aerial photograph taken with the UAV.

The map partially shows the study area along the river (each one of the 18 black dots represent a study site; 90 sites were sampled in total).


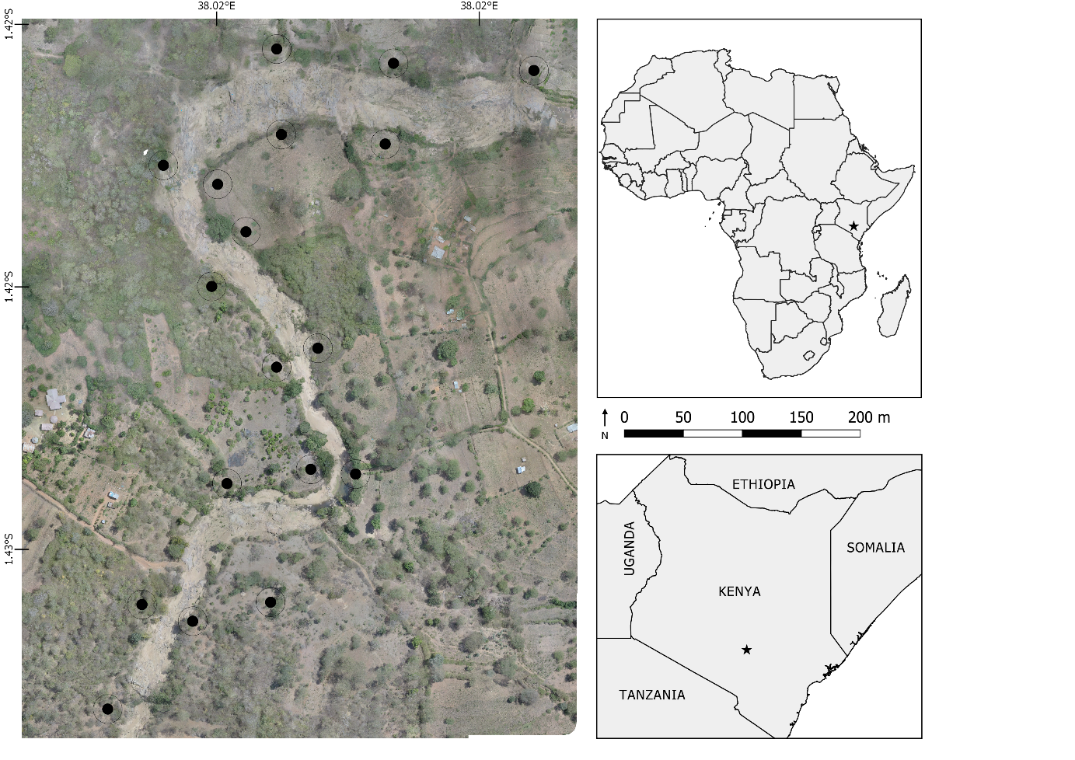

Supplement: Supplementary file 1 — Appendix S1 [file ECE3-11-12665-s001.docx]
